# The Roles of Noncoding RNAs in Systemic Sclerosis

**DOI:** 10.3389/fimmu.2022.856036

**Published:** 2022-04-08

**Authors:** Yongmei Liu, Linlin Cheng, Haoting Zhan, Haolong Li, Xiaomeng Li, Yuan Huang, Yongzhe Li

**Affiliations:** ^1^ Department of Clinical Laboratory, Peking Union Medical College Hospital, Peking Union Medical College and Chinese Academy of Medical Sciences, Beijing, China; ^2^ State Key Laboratory of Complex, Severe and Rare Diseases, Peking Union Medical College Hospital, Chinese Academy of Medical Science and Peking Union Medical College, Beijing, China; ^3^ Department of Medical Research Center, Peking Union Medical College Hospital, Chinese Academy of Medical Science and Peking Union Medical College, Beijing, China

**Keywords:** ncRNA, miRNA, lncRNA, systemic sclerosis, function, biomarker

## Abstract

Noncoding RNAs (ncRNAs) constitute more than 90% of the RNAs in the human genome. In the past decades, studies have changed our perception of ncRNAs from “junk” transcriptional products to functional regulatory molecules that mediate critical processes, including chromosomal modifications, mRNA splicing and stability, and translation, as well as key signaling pathways. Emerging evidence suggests that ncRNAs are abnormally expressed in not only cancer but also autoimmune diseases, such as systemic sclerosis (SSc), and may serve as novel biomarkers and therapeutic targets for the diagnosis and treatment of SSc. However, the functions and underlying mechanisms of ncRNAs in SSc remain incompletely understood. In this review, we discuss the current findings on the biogenetic processes and functions of ncRNAs, including microRNAs and long noncoding RNAs, as well as explore emerging ncRNA-based diagnostics and therapies for SSc.

## Introduction

### Systemic Sclerosis

Systemic sclerosis (SSc) is a rare, systemic autoimmune disease characterized by vascular damage, immune activation, and fibrosis of the skin and/or internal organs (1). The estimated prevalence of SSc was 88 per million in men and 514 per million in women, demonstrating sex-based differences (2). Patients with SSc are divided into different categories according to their skin condition: limited cutaneous SSc (lcSSc, skin fibrosis is restricted to the fingers, distal extremities, and face), diffuse cutaneous SSc (dcSSc, skin fibrosis is present in the trunk and proximal extremities), SSc sine scleroderma (no detectable skin involvement), and overlap syndrome (concomitant with another connective tissue disease) (3, 4). The definitive diagnosis of SSc is usually based on the 2013 European League Against Rheumatism (EULAR) and American College of Rheumatology (ACR) classification criteria (5, 6). Some key early clinical characteristics of SSc are associated with early internal organ involvement; however, there are no reliable criteria to identify patients with early symptoms. Thus, novel biomarkers and therapeutic targets of SSc are needed to improve its diagnosis and prognosis. Notably, numerous studies have reported that noncoding RNAs (ncRNAs), such as microRNAs (miRNAs) and long noncoding RNAs(lncRNAs), are implicated in the development of SSc.

### ncRNAs

ncRNAs constitute more than 90% of the RNAs in the human genome. Scientists have discovered >50,000 ncRNAs in the past decade, but most remain unstudied (7). Unlike messenger RNAs (mRNAs), which transmit instructions encoded in the DNA for proteins production, ncRNAs are not involved in protein production (8). ncRNAs include intronic RNAs (9), miRNAs (10), lncRNAs (11), circular RNAs (12), extracellular RNAs (13), transfer RNA-derived small RNAs (14), and piwi-interacting RNAs (15). Although ncRNAs cannot encode proteins, they can modulate the expression of other genes through a variety of mechanisms at transcriptional and post-transcriptional levels, thereby affecting both normal cellular function and disease development. Studies on ncRNAs in SSc have focused on miRNAs and lncRNAs. This review summarizes the latest research on miRNAs and lncRNAs including their biosynthesis and functions, as well as their roles in SSc and its complications and as potential biomarkers.

## Biosynthesis and Functions of miRNAs and lncRNAs

### miRNA

**Figure 1:**
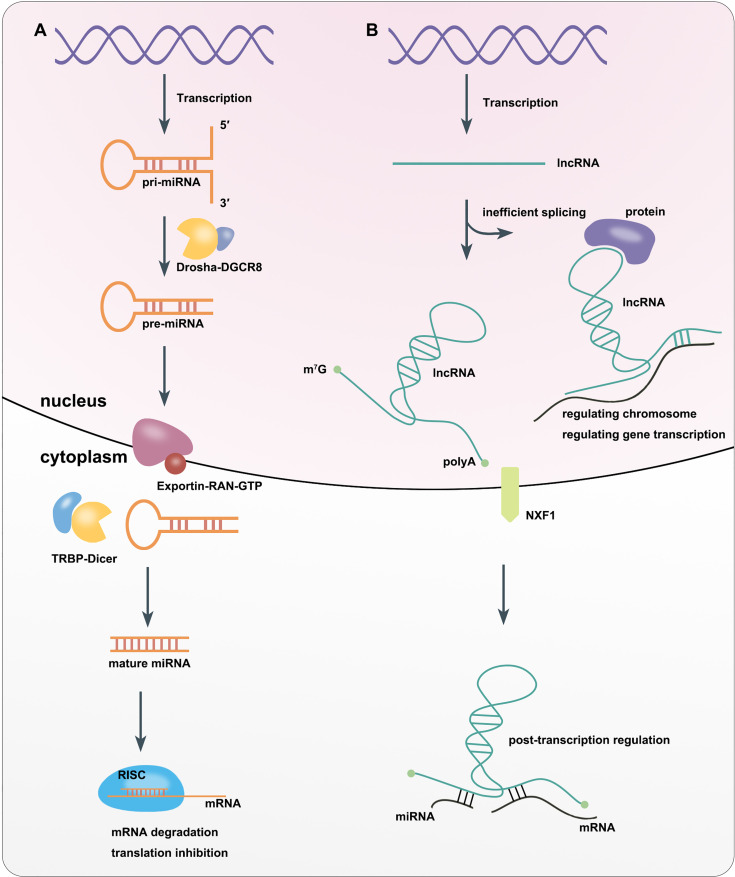
Biosynthesis and Function of miRNA **(A)** and lncRNA **(B)**.

miRNAs are is a class of ncRNAs of that are 21–25 nucleotides (nt) in length. They were first discovered in *Caenorhabditis elegans* in 1993 (16). miRNAs can be divided into intracellular miRNAs, and extracellular miRNAs or circulating miRNAs (17). RNA polymerase II or III transcribes miRNA-related genes to primary miRNAs (pri-miRNAs) with thousands of nt in the nucleus. The core part of pri-miRNAs is a stem loop structure, whose double-stranded stem region contains functional miRNAs (18, 19). Then Drosha, a type III RNA cutting enzyme, forms a complex with DiGeorge syndrome critical region gene 8 (DGCR8), a double-stranded RNA-binding protein that recruits pri-miRNA substrates, which subsequently cleaves the pri-miRNA into pre-miRNA with only 60–70 nt. This leads to the formation of a hairpin structure (18, 20). Next, the Exportin5–RAN–GTP complex exports pre-miRNA from the nucleus into the cytoplasm (18, 21). In the cytoplasm, where the enzyme RNase Dicer enzymes, which binds to transactivation response element RNA-binding protein (TRBP), cleaves the pre-miRNA into mature-length miRNAs while the miRNA is still double-stranded (18, 22). One strand of the mature miRNA (the guide strand) interacts with Argonaute (AGO) proteins to enter the RNA-induced silencing complex (RISC). Finally, RISC induces target mRNA degradation or inhibiting gene translation by sequence complementary binding (23, 24) (**Figure 1A**).

### lncRNA

lncRNAs are defined as RNAs longer than 200 nt, which are not translated into functional proteins. lncRNAs are often capped by 7-methylguanosine (m^7^G) at their 5′ ends, polyadenylated at their 3′ ends, and spliced like mRNAs. But unlike mRNAs, many transcribed lncRNAs are retained in the nucleus due to inefficient processing by RNA polymerase II (Pol II), whereas others are spliced and exported to the cytoplasm by nucleus RNA export factor 1 (NXF1) (25–27). lncRNAs localized in the nucleus can interact with DNA, RNA, proteins, and other molecules to regulate the structure and function of chromosomes and cis–trans gene transcription as well as influence mRNA splicing, stability and translation. lncRNAs in the cytoplasm mostly trans-regulate gene expression at the post-transcriptional level, including regulating mRNA translation and degradation or intracellular signaling pathways. In addition, some lncRNAs can localize to organelles and regulate their function and metabolism, such as mitochondrial oxidation and homeostasis (28) (**Figure 1B**).

## ncRNAs in SSc

SSc is a chronic, progressive multifocal disease characterized by vascular alterations, autoimmunity, and fibrogenesis caused by environmental factors in genetically susceptible individuals (1). Its earliest stages feature microvascular injury and endothelial cell activation (29). Altered endothelial cells and infiltrating immune cells interact to secret proinflammatory cytokines and prefibrotic mediators, leading to myofibroblast transition and excessive extracellular matrix (ECM) deposition, culminating in fibrosis (30–32). Dysregulated miRNAs and lncRNAs in SSc tissues and blood act as inducers or suppressors, correlating with vascular changes, immune activation, and fibrosis. Herein, we summarize recent studies on the role of miRNAs (**Table 1**) and lncRNAs (**Table 2**) in SSc development.

**Table 1:** The Role of miRNAs in SSc.

	miRNA	Function	Tissue and cell	Expressed in SSc	reference
Microvascular changes	miR-22	Promoted proliferation and migration, neointima formation by increasing target genes expression.	VSMCs	down	(33)
	miR-193b	Promoted VSMCs proliferation and inhibited apoptosis by over-expressing uPA.	fibroblasts	down	(34)
Immune activation	miR-155	Synergized with the activation inflammasome to increase collagen synthesis by promoting IL-1 release.	fibroblasts	up	(35)
		Promoted lung macrophages alternative activation, controlled toll-like receptor signaling, and associated with profibrotic gene expression, lung function tests.	lung, PBMCs	up	(10, 36)
	miR-26b-5p	Promoted cells fibrosis markers expression by regulating NF-κB or JAK-STAT pathway.	fibroblasts	up	(37)
	miR-21	Controlled toll-like receptor signaling and correlated with several profibrotic genes.	fibroblasts	up	(10, 36, 38, 39)
	miR-618	Eradicated circulating DCs by degrating IRF-8 and induced autoimmunity by producing IFNα.	pDC	up	(40)
	miR-126	Caused SSc-pDC imbalance by upregulating IFN-inducing genes and targeting some signal pathway.	pDC	up	(41)
	miR-139-5p	Caused pDC dysregulation by upregulating IFN-inducing genes and targeting some signal pathway, promoted type I IFN response and autoimmune response by USP24.	pDC	up	(41)
Fibrosis	miR-483-5p	Promoted ECM and myofibroblast phenotype by regulating fibrosis-related genes expression.	Fibroblasts, endothelial cells	up	(42)
	miR-3606-3p	Inhibited cell cycle progression, p-SMAD2/3 and collagen production and TGF-β pathway by binding to 3′-UTR of TGFBR2.	dermal tissues, fibroblasts	down	(43)
	miR-155	Enhanced fibrosis-associated Wnt/β-catenin and Akt pathways by binding and decreasing CK1α and SHIP-1 protein, associated with the extent of skin sclerosis.	skin tissue	up	(44)
	miR-16-5p	Promoted fibroblast activation by inducing expression and activating NOTCH signaling.	skin, fibroblasts	down	(45)
	miR-130b	Promoted fibrosis-related genes expression and TGF- β signaling through binding to PPAR γ	skin tissues, fibroblasts	up	(46)
	miR-202-3p	Increased profibrotic expression and collagen deposition in primary skin fibroblasts by directly regulating MMP1	skin tissues, fibroblast	up	(47)
	miR-320a	Inhibited collagen expression by combining and inhibiting TGFBR2 and IGF1R.	PBMCs, lung	down	(48, 49)
Therapy target	miR-26a-2-3p	Blocked IFN signaling transport by inhibiting some ISGs expression.	monocytes	down	(50)
	miR-151-5p	Prevented osteopenia, skin tighten and immune disorders by inhibiting the IL-4Rα/mTOR pathway.	serum exosomes of MSCs	down	(51)
	miR-5196	Inhibited Fra2 and TIMP-1 expression.		down	(52)
	let-7, miR-99a, miR-125b	Inhibited ECM deposition, dermal thickening, and inflammatory infiltration by regulating the TGF β and WNT signaling pathways	BMSC-EVs	up	(53)
	miR-21	Inhibited cell apoptosis and promoted survival by targeting 3’-UTR of Bcl-2 mRNA.	fibroblasts	up	(54)
	miR-125b	Promoted cell apoptosis and decreased proliferation by increasing BAK1, BMF and BBC3 expression.	skin, fibroblasts	down	(55)

**Table 2:** The Role of lncRNAs in SSc.

	lncRNA	Function	Tissue and cell	Expressed in SSc	reference
Microvascular changes	SMILR	Promoted cells proliferation by interacting with CENPF mRNA and STAU1.	VSMCs	up	(56)
	OTUD6B-AS1	Inhibited cells proliferation and suppressed apoptosis, led to cell dysregulation on the fibrosis and microangiopathy by increasing Cyclin D1 expression,	fibroblasts,HPASMCs	down	(57)
Immune activation	NRIR	Destroyed autoinflammation homeostasis by inducing IFN response, associated with severe clinical phenotypes.	monocytes	up	(11)
	PSMB8-AS1	Promoted cells activation and proinflammatory cytokines secretion.	monocytes	up	(58)
Fibrosis	H19X	Promoted ECM production, myofibroblast activation and inhibiting cells apoptosis by binding to DDIT4L.	fibroblasts, skin, lung	up	(59)
	HOTAIR	Promoted cell profibrotic activation by inducing H3K27me3 in miR-34a.	skin, fibroblasts	up	(60, 61)
	TSIX	Promoted fibrosis by enhancing collagen mRNA stability and expression.	fibroblasts	up	(62)

### Microvascular Changes

Microvascular injury and endothelial cell activation are the earliest and possibly primary events of SSc (5). Vascular injury leads to a decrease in the number of capillaries, proliferation of intimal and smooth muscle cells, thickening of the vascular wall, and narrowing of the lumen, consequently resulting in tissue hypoxia and oxidative stress (5). In addition, activated endothelial cells promote the expression of adhesion molecule, leading to the recruitment of inflammatory cells and secrete profibrotic factors [e.g., endothelin 1 and connective tissue growth factor (CTGF)] and profibrotic mediators [e.g., TGFβ, platelet-derived growth factor (PDGF), interleukin (IL)-1, and IL-6], which stimulate vascular smooth muscle cell (VSMC) proliferation and ECM production (5).

#### miRNA

miR-22 has been shown to modulate the expression and phenotype of VSMCs in human femoral arteries, and its overexpression reduces the proliferation and migration of VSMC, formation of neointima by binding to 3′ UTRs of target genes (*MECP2, HDAC4, and EVI*1) and degrating them (33). miR-193b-3p (miR-193b) was downregulated in fibroblasts and skin biopsy samples of patients with SSc but was unaffected by major profibrotic cytokines and hypoxia. Additionally, miR-193b inhibition resulted in the overexpression of its target, urokinase-type plasminogen activator (uPA), which was strongly expressed in VSMCs in SSc skin section and induced proliferation and inhibited the apoptosis of human pulmonary artery smooth muscle cells (HPSMCs) in an uPAR-independent manner. This led to the proliferative vasculopathy with intimal hyperplasia characteristic in SSc. Such vascular changes reduced blood flow and contributed to pulmonary arterial hypertension. Therefore, miR-193b, as a post-transcriptional regulator of uPA, could be a therapeutic target for SSc-related vasculopathy (34).

#### lncRNA

Smooth muscle enriched lncRNA (SMILR) is enriched in VSMCs; this recently discovered intergenic lncRNA specifically targets the late mitotic pathway in proliferating VSMCs by interacting with centromere protein F (CENPF) mRNA and STAU1 (56). CENPF controls mitosis, transcriptional regulation, and muscle cell differentiation (63). SMILR knockdown has been shown to silence CENPF, leading to the accumulation of binucleated cells and reduced proliferation (56). Other studies have indicated that STAU1 overexpression affects mitotic entry and impairs the proliferation of transformed cells (64). SMILR and STAU1 reportedly interact to inhibit STAU1 function during the cell cycle, thereby regulating the proliferation of VSMCs. Thus, SMILR could be a novel target for the treatment of aberrant growth of VSMCs (56). Antisense(AS) lncRNAs are transcribed from the opposite strand of protein-coding genes and overlap with one or more exons and introns of the gene’s sense. The expression of most AS lncRNAs is approximately 10-fold lower than that of their coding gene and is more tissue-specific (65–67). AS lncRNAs not only regulate sense gene expression but also act independently of the sense gene (7). lncOTUD6B-AS1 was significantly downregulated in the involved skin tissues compared with noninvolved skin tissues of patients with SSc. OTUD6B-AS1 knockdown significantly reduced proliferation in both dermal fibroblasts and HPASMCs by increasing target cyclin D1 expression at the mRNA and protein levels. It’s report that overexpression of cyclin D1 prevented DNA repair and prevented cells from entering in S phase (68). In addition, OTUD6B-AS1 knockdown favored cells apoptosis resistance, which can be regarded as a compensatory mechanism for inhibition of cells proliferation. Therefore, downregulated OTUD6B-AS1 resulted in cell dysregulation in SSc-related fibrosis and microangiopathy (57).

### Immune Activation

Dysregulation of both innate and adaptive immunity may promote SSc. Signs of immune activation in SSc include the presence of inflammatory cells and inflammatory signatures in organs such as the skin and lungs, changes in the number and function of circulating immune cells, presence of type I interferon (IFN) signature in immune cells; and presence of serum autoantibodies. Polymorphisms of IFN regulatory factor 5 (IRF5) and signal transducer and activator of transcription 4 (STAT4), as well as several other immune pathway genes are linked to SSc (69).

#### miRNA

miR-155 regulates innate and adaptive immune responses (70, 71). Artlett et al. have showed that NLRP3 inflammasomes can enhance IL-1 transcription and promote autocrine signaling, further driving miR-155 expression. Then miR-155 synergizes with the inflammasome to induce a positive feed-forward signal that further promotes IL-1 release, leading to continual collagen expression (35). Bioinformatics analyses revealed that miR-26b-5p functions in fibroblasts and targets CXCL9 and CXCL13 (37). An miR-26b-5p inhibitor was previously reported to promote the activation of negative regulators of the NF-κB and JAK-STAT pathway, thereby hindering the expression of CXCL9 and CXCL13 and subsequently decreasing the mRNA levels of two known fibrosis markers, α-SMA and fibroblast activation protein (FAP), as well as the collagen-type I alpha 2 (Col1A2) and collagen-type IV alpha 1 (Col4A1), consequently impeding fibrosis (37, 72). In addition, Christmann et al. found dysregulated miRNAs in lung and in peripheral blood mononuclear cells (PBMCs) in SSc with interstitial lung disease (SSc-ILD) patients. For example, miR-155, which is highly expressed in SSc-IDL patients, is associated with profibrotic gene expression (*SPP1* and *POSTN*) and lung function test findings, such as high-resolution computed tomography lung score, forced vital capacity, and diffusing capacity of the lung for carbon monoxide. However, miR-155 knockdown blocked the alternative activation of lung macrophages, leading to longer survival and less aggressive lung fibrosis in mice model (10). Increased miR-21 in SSc-IDL lung fibroblasts also correlates with the upregulation of several profibrotic genes, such as *Col3a1* and *POSTN* (10). miR-21 along with miR-155 control toll-like receptor signaling (38), suggesting that they regulate immune activation in SSc-ILD.

In SSc, dysregulation of the immune system precedes effects on skin and lungs. Lung inflammation correlates with progressive lung fibrosis marked by TGFβ (73). miRNAs are a class of regulators that tightly control the overall inflammatory response. Rossato et al. reported the upregulation of miR-618 in plasmacytoid dendritic cells (pDCs) of patients with SSc, which led to the degradation of its target IFN regulatory factor 8 and consequently the eradication of circulating DCs (40), resulting in severe immunodeficiency in the affected patients (74, 75). Furthermore, miR-618 induced pDCs to produce a greater amount of IFNα in response to toll-like receptor 9 stimulation, contributing to the type I IFN signature observed in SSc patients. Such early molecular changes in the pDCs of patients with SSc suggested that miR-618 was an important epigenetic target to regulate immune system homeostasis and had important effects in the immune system and induce autoimmunity (40). Similarly, Chouri et al. showed that miR-126 and miR-139-5p were increased in pDC of patients with SSc in the earliest stage. Their expression upregulated IFN-inducing genes as well as IFIT3, IFI6, IFIT1, and CXCL10, which activate pDCs and trigger SSc *via* TLR9-mediated response and IFN signaling. Both miRNAs mainly target the PDGF, insulin-like growth factor, vascular endothelial growth factor, and IL signaling pathways, which also contribute to SSc–pDC imbalance. Further, miR-139-5p inhibited the expression of the deubiquitinating enzyme USP24 (41), which negatively regulates type I IFN response and autoimmune response (76). Altogether, the elevated levels of miR-126 and miR-139-5p might reflect the activation of circulating pDCs in SSc.

#### lncRNA

Monocytes of patients with lcSSc and noncutaneous SSc (ncSSc) showed higher expression of lncNRIR. As an IFN-dependent lncRNA, NRIR positively regulates LPS-induced IFN response in human monocytes. Silencing NRIR suppresses the IFN-stimulated genes *CXCL10* and *CXCL11* (11), which are linked to type I IFN signature, more severe clinical phenotypes, and lung and kidney involvement (77–79). Knocking down NRIR blocks IFN receptor signaling in SSc, maintaining autoinflammation homeostasis (11). LncPSMB8-AS1 is also upregulated in the blood monocytes of patients with early SSc, ncSSc and dsSSc and is implicated in immune cell activation and vesicle-related transport. PSMB8-AS1 knockdown resulted in decreased concentrations of IL-6 and TNFα in the cell-free supernatant (58), which are linked to fibrotic (80, 81), and ILD (82, 83). Thus, PSMB8-AS1 can modulate monocyte activation in SSc and secretion of proinflammatory cytokines (58).

### Fibrosis

The accumulation of a fibrous matrix composed of collagen, elastin, glycosaminoglycan, and fibronectin is a hallmark of SSc. Activated stromal cells increasing synthesis, prolyl and lysyl oxidase and transglutaminase 2 enhancing assembly and crosslinking catalysed, as well as defective degradation together result in excessive accumulation of ECM during fibrosis. This process replaces normal tissue architecture with connective tissue, leading to permanent scarring (84, 85). α-SMA-positive and apoptosis-resistant myofibroblasts in fibrotic tissue secrete matrix molecules, TGFβ, and other profibrotic mediators, promoting ECM accumulation and remodeling (86).

#### miRNA

miR-483-5p is upregulated in localized scleroderma and SSc (from the early stages of the disease onwards) but not in other systemic autoimmune diseases, indicating that this miRNA could be linked to scleroderma-specific fibrosis. The overexpression of miR-483-5p in fibroblasts and endothelial cells regulates the expression of fibrosis-related genes, such as *COL4A1* and *COL4A2* encoding for collagen IV (42), a primary collagen in the basement membrane surrounding blood vessels and in the dermoepidermal junction in the skin that positively correlates with the modified Rodnan skin score (mRSS) (87). On the other hand, high miR-483-5p levels in endothelial cells enhance the transcriptional levels of α-SMA and SM22A (two myofibroblast differentiation markers), which promote the myofibroblast phenotype. In addition, miR-483-5p disrupts collagen homeostasis by downregulating Fli-1 and promoting ECM production. Serum exosome samples obtained from patients with SSc have high levels of miR-483-5p, which may contribute to the disease’s fibrotic phase (42). In a study by Shi, the levels of miR-3606-3p were significantly reduced in dermal tissues and primary fibroblasts. miR-3606-3p may function as a novel antifibrotic miRNA by targeting TGF-βreceptor types 2 (TGFBR2): overexpressed miR-3606-3p could significantly decrease the mRNA and protein levels of TGFBR2 by targeting its 3′-UTR, consequently reducing the production of p-SMAD2/3 and type I collagen as well as hindering cell cycle progression and TGF-βassociated signaling (43). The TGF-β pathway promotes fibrosis in SSc by modulating proliferation, activation, and accumulation of fibroblasts and stimulating ECM production (88). Upon stimulation, TGFBR2 first binds to TGF-β, autophosphorylates, and subsequently activates TGFBR1 to trigger a cascade response that regulates a series of fibrosis-related factors, such as collagens, α-SMA, CTGF, MMPs, tissue inhibitor of metalloproteinases (TIMPs), and fibronectin (89–92). Elevated miR-155 expression in skin tissue from SSc patients and mice model, which corresponded to the extent of skin sclerosis, and silencing miR-155 could inhibit fibrosis-associated Wnt/β-catenin and Akt pathways by upregulating the protein levels of CK1αand SHIP-1. In addition, antagomiR-155 treatment effectively reduced bleomycin-induced skin fibrosis in mice, suggesting that miR-155 is a potential therapeutic target for scleroderma (44). Another study reported reduced miR-16-5p expression in patients with SSc compared with healthy controls. miR-16-5p knockdown induced NOTCH2 mRNA expression and activated NOTCH signaling in human skin fibroblasts by hindering binding to NOTCH2 mRNA for degradation, which promoted the expression of α-SMA, Col 1α, and CTGF and inhibited the expression of collagenases MMP1 and MMP8. Indeed, miR-16-5p suppressed myofibroblast activation by decreasing NOTCH signaling and may prove promising in clinical treatments (45). miR-130b was upregulated in the dermis skin biopsy samples of patients with SSc, fibroblasts and skin tissues of fibrosis-mice model. miR-130b increased expression of fibrosis-related genes *COL1A1*, *COL1A2*, *α-SMA* and *Fn*, enhancing TGF- β signaling by negatively regulating PPARγ, an antifibrotic molecule (46). Similarly, miR-202-3p accumulated in skin tissues of patients with SSc and primary fibroblast compared with normal tissues and increased profibrotic gene expression as well as collagen deposition in primary skin fibroblasts by directly regulating MMP1 (47), which is involved in ECM degradation (93). miR-320a plays an antifibrotic role in SSc-ILD. PBMCs obtained from patients with SSc-ILD as well as lung tissues obtained from bleomycin-induced SSc-ILD mouse models showed reduced levels of miR-320a, which negatively regulates the expression of collagen genes by directly binding to and inhibiting TGFBR2 and IGF1R (48). TGFBR2 is a transmembrane serine/threonine kinase that forms a heterodimeric complex with TGFBR1, which can bind to TGF-β, which subsequently phosphorylates downstream proteins that promote fibrosis (94). IGF1R is the respective receptor of IGF1 signaling pathway, associating with pathological fibrosis states (95, 96).

#### lncRNA

LncRNA H19X is a key mediator of matrix remodeling in fibroblasts and SSc-related cell types during the fibrotic phase of SSc. Indeed, H19X is upregulated in the skin of patients with SSc when compared with that of HCs. Lung samples of patients with SSc-ILD also show H19X upregulation. This overexpression is not surprising considering that H19X is related to the TGF-β pathway, and TGF-β can promote H19X expression in a time- and dose-dependent manner in SSc-related cell types. The activated TGF-β pathway induces H19X expression, which promoted ECM production, myofibroblast activation and inhibited fibroblast apoptosis. As a pathogenic factor in SSc, H19X inhibits DNA damage-inducible transcript 4-like protein (DDIT4L) expression through the direct contact of H19X RNA with a DNA regulatory element upstream of the *DDIT4L* (59). DDIT4L inhibits ECM production under normal conditions and may be useful for predicting radiation-induced fibrosis (97). H19X acts as a key effector of TGF-β-induced ECM remodeling and fibrosis (59). Fibroblasts and skin tissues of patients with SSc showed high expression of lncHOTAIR, a potential factor involved in SSc development that drives the profibrotic activation of dermal fibroblasts *via* the enhancer of zeste 2 (EZH2)/miR-34a/NOTCH/collagen/α-SMA pathway. HOTAIR directs modulates EZH2 to induce H3K27me3 of miR-34a, thereby inhibiting miR-34a expression (60), which consequently suppresses NOTCH (98). This results in increased levels of collagen and α-SMA *in vitro* and *in vivo* (60). Other studies also showed that HOTAIR could activate dermal fibroblasts *via* the EZH2/miR-34a/NOTCH/GIL2 pathway. Upregulated HOTAIR caused NOTCH inhibition by EZH2/miR-34a in above mechanism, leading to GLI2 transcription, which promoted profibrotic markers (*collagen*, *α-SMA*, *CTGF*) expression (61). Dermal fibroblasts upregulate another downstream target of TGF-β, lncTSIX. TSIX knockdown was previously reported to reduce reduced the expression of type I collagen mRNA, which hindered SSc-related fibrosis (62). Messemaker et al. have also found that the expression levels of AS lncRNAs, including CTBP1-AS2, AGAP2-AS1, and OTUD6B-AS1, in SSc patients skin tissues showed variations: CTBP1-AS2 and AGAP2-AS1 increased, whereas OTUD6B-AS1 decreased. These three AS lncRNAs are related to their coding genes, indicating that they play functional roles in SSc pathogenesis (99).

**Figure 2:**
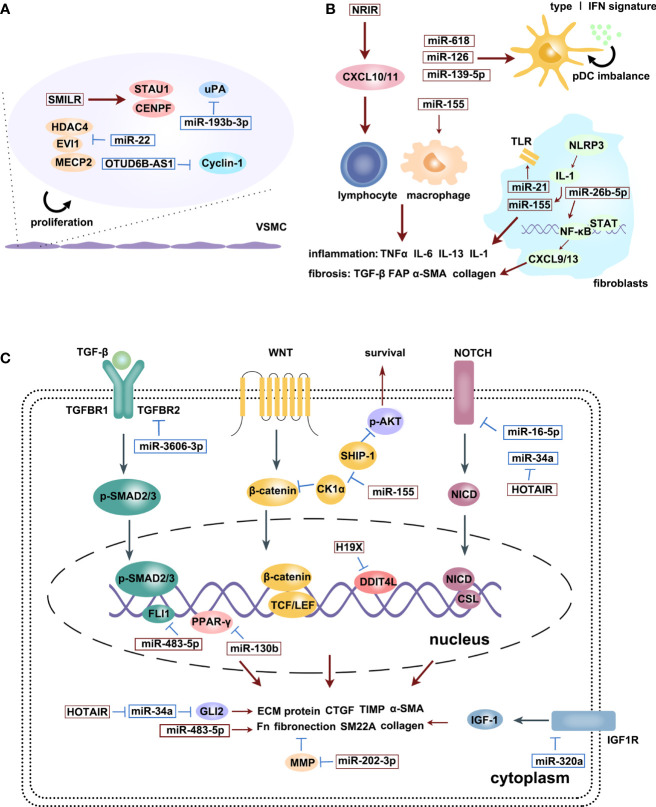
The regulatory effects of various miRNAs and lncRNAs involved in SSc process: microvascular changes **(A)**, immune activation **(B)** and fibrosis **(C)**. Upregulation (red squares) and downregulation (blue squares) of diverse ncRNAs participate in different signaling pathways, promoting SSc progression. Red arrows are promotion symbols, blue lines are inhibition symbols.

**Figure 2** is an illustration of the regulatory effects of various miRNAs and lncRNAs involved in SSc.

## miRNAs as Therapeutic Targets in SSc

The ability to regulate multiple genes enables miRNAs to promote cancer and autoimmune diseases (36, 39). To treat such conditions, miRNAs could be used as therapeutics (miRNA mimics) or as targets of therapeutics (anti-miRs) (49), which rely on the natural RNAi mechanism of miRNAs in cells. miRNAs only need to be partially complementary to their target mRNAs to exert its post-transcriptional and translational inhibitory effects, thereby achieving the therapeutic silencing of multiple target genes (100). The expression of miR-26a-2-3p is lower in the monocytes of patients with SSc than in those of HCs. Exogenous delivery of miRNA-26a-2-3p to TLR-stimulated monocyte can inhibit some IFN-stimulated genes, such as *IRF6*, *GBP2*, *IFI44L*, *Siglec1*, and *IFN-β*, thereby blocking IFN signal transduction in SSc development. These findings support the use of miRNA therapeutics as a novel therapy for SSc (50). The serum of patients with SSc showed decreased levels of miR-151-5p. Deriving from exosomes of mesenchymal stem cells, miR-151-5p determined osteogenic versus adipogenic fate and rescued the osteopenia phenotype in Tsk/+ mice by inhibiting the IL-4Rα/mTOR pathway (51). Low levels of IL-4 or IL-4Rα can inhibit the fibrosis and osteopenia phenotypes (101, 102). Systemic delivery of miR-151-5p also prevented osteopenia, skin tightening, and reduced T_H_2 cell differentiation and T-cell infiltration in the hyperdermal area, indicating that miR-151-5p-based gene therapy could achieve therapeutic effects in multiple organs in Tsk/+mice. Considering that miR-151-5p present in mice and humans is 100% identical, it could be a suitable target for SSc treatment (51). The exogenous transfection of miR-5196 significantly reduced the expression of Fos-related antigen 2 (Fra2, AP-1 transcription factor) by binding to its five seed regions within the 3′-UTR and indirectly inhibited TIMP-1, indicating its potential suppression in SSc (52).

On the other hand, miRNAs in exosomes can also be an effective therapy method for SSc. Exosomes are microvesicles with lipid bilayers, containing miRNAs, proteins, metabolites, and other molecules, which can be secreted by cancer cells and a variety of stromal cells. After secretion, exosomes transduce endocrine, paracrine and autocrine signals and can mediate intercellular communication by releasing their contents into target cells through fusion (103). Remarkably, bone marrow mesenchymal stem cell-derived extracellular vesicles (BMSC-EVs), which contain several highly expressed miRNAs such as let-7 family, miR-99a, and miR-125b, were reported to reduce bleomycin-induced dermal thickening and fibrosis as well as abnormal deposition of collagen in a mouse model of SSc. BMSC-EVs treated SSc mice have decreased TGF-β1-positive cells,α-SMA-positive myofibroblasts, mast cells and infiltrating macrophages as well as lower mRNA levels of the inflammatory cytokines IL6, IL10, and TNF-α. BMSC-EVs might be useful for treating SSc using miRNAs, which could alleviate ECM deposition and inflammatory cell infiltration by regulating the TGFβ and Wnt signaling pathways (53). A previous study reported that there are 6 profibrotic miRNAs and 10 antifibrotic miRNAs in the serum exosomes of patients with SSc; further, the levels of 8 existing miRNAs were significantly different between the exosomes of patients with limited and diffuse SSc. The serum exosomes of patients with SSc patients stimulated normal fibroblast expressing of ECM components, such as Col 1A1, Col 3A1, FN1, and also induced the expression of three myofibroblast-associated genes *COMP*, *α-SMA*, and *FN-EDA* in dose-dependent manner. These findings suggest that exosomes containing miRNAs release their profibrotic contents upon fusion with target cells in their extracellular environment (104).

Apoptosis refers to the endogenous programmed cell death that maintains homeostasis (105). The inhibition of apoptosis is a common hallmark of several diseases, such as cancer (106), fibrosis (107), and autoimmune diseases (108). As is known, fibroblasts are the central mediators of fibrotic manifestations in SSc. In SSc, fibroblasts in lesional areas, which are mostly myofibroblasts, secrete ECM, resulting in excessive scarring of the skin (109). The decreased susceptibility of fibroblasts to apoptosis drives fibrosis in SSc and represents a potential therapeutic target (107). Farsangi et al. showed that miR-21 upregulation enhanced Bcl-2 expression in both SSc and normal fibroblasts (54) by directly targeting Bcl-2 mRNA at the 3′-UTR region (110), thereby preventing apoptosis and promoting cell survival in SSc fibroblasts (54). Another study showed that miR-125b is downregulated in the skin and primary dermal fibroblasts of patients with SSc. Further, miR-125b knockdown decreased the number of α-SMA-positive cells, increased apoptosis, and decreased the proliferation of dermal fibroblasts by increasing the expression of BAK1, BMF, and BBC3, which are involved in the mitochondrial apoptotic pathway. Thus, miR-125b may act as a disease suppressor, exerting compensatory protective effects directed against excessive skin fibrosis (55).

## As biomarkers in SSc

Early detection and treatment are key to improving SSc prognosis. As miRNAs and lncRNAs mainly function upstream of various signaling pathways, making them promising diagnostic tools. miRNAs can be released into the extracellular environment by binding to RNA-binding proteins or by secretion from cell-derived plasma exosomes (111–113). Extracellular miRNA expression levels can reflect one’s physiological state, as indicated by the profiles of dysregulated miRNAs in plasma and serum (112, 114). Recent developments in next-generation sequencing have made profiling miRNA levels in biological fluids a viable approach to discover biomarkers (115). Additionally, miRNAs in peripheral blood are stable and resistant to degradation by ribonucleases in the body fluids (112). Thus, miRNAs in blood represent promising new complementary biomarkers for SSc. However, currently, there are few studies on lncRNAs as biomarkers of autoimmune diseases, with only one study on SSc. Herein, we summarized the recent findings on miRNAs and lncRNAs as biomarkers present in the blood of patients with SSc (**Table 3**).

**Table 3:** ncRNAs as biomarkers in SSc.

miRNA	Expressed in blood	Clinical correlation	AUC, sensitivity, specificity	Reference
miR-483-5p	up	Correlated with mRSS in dcSSc patients	0.815, 0.656, 0.821	(85, 89)
miR-155	up in skin	Correlated with extent of skin sclerosis in SSc		(91)
miR-146a	down	Correlated with lung fibrosis, skin involvement degree and sex.		(116)
miR-5196	up	Correlated with the CRP levels.		(52)
miR-618	up	Correlated with disease progression and the presence of ILD.		(40)
let7b and -7d	down in skin	Negatively correlated with severity of PH.		(117)
**LncRNA**				
ANCR	down	Correlated with mRSS, disease duration, PH and altered lipid profile.	0.63, 0.5079, 0.6857	(118)
TINCR	up	Correlated with ESR.	0.64, 0.6825, 0.5429	(118)
HOTTIP	up	Correlated with mRSS.	0.67, 0.5161, 0.7143	(118)
SPRY4-IT1	up	Correlated with mRSS and PH.	0.76, 0.7581, 0.6571	(118)
SPRY4-IT1	up	Higher in dcSSc than lcSSc.	0.68, 0.7368, 0.619	(118)
TSIX	up	Correlated with the greater dcSSc: lcSSc ratio and higher mRSS.		(62)

### miRNA

The circulating serum miR-483-5p and snRNA-U6 show elevated expression levels in all SSc disease subsets (early SSc, ncSSc, dcSSc, and lcSSc). Their expression levels are particularly correlated with mRSS in patients with dcSSc. Receiver operating characteristic curve (ROC) analysis revealed the high predictive ability of miR-483-5p as a biomarker of SSc, with area under the curve, sensitivity, and specificity of 0.815, 0.656, and 0.821, respectively. More than 60% of SSc patients have miR-483-5p levels exceeding the potential optimal cut-off of normal levels (89). Vreca et al. showed that miR-146a relates to the predisposition and phenotypic heterogeneity of SSc, namely the higher prevalence of miR-146a rs2910164 C > G genotype in patients with SSc having lung fibrosis and an active form of the disease. In addition, miR-146a was downregulated in PBMCs of patients with SSc compared with those of HCs, and the levels of miR-146a were lower in women than in men. Patients with SSc showing mild skin involvement (mRSS score ≤ 10) expressed 36% less miR-146a than those with severe skin involvement. Altogether, reduced miR-146a expression represents a risk factor for the development and progression of SSc (116). Another study reported elevated circulating miR-5196 in the serum and monocytes of patients with SSc, which were positively correlated with C-reactive protein levels. This suggests miR-5196 can be used as a biomarker of inflammation in SSc (52). Rossato’s study showed that miR-618 is incrementally upregulated in pDCs of patients with early SSc (fold change, 1.35), SSc patients without fibrosis (fold change, 1.41), and patients with overt fibrosis (fold change, 3.85), including those with dcSSc with a disease duration of ≤2 years (fold change, 3.68). Higher levels of miR-618 were also associated with ILD (40). Rusek et al. found that miR-4484 was also upregulated up to 18-folds in the serum of patients with SSc compared with that of HCs (119).

Pulmonary hypertension (PH) is one of the most serious complications in patients with SSc. A meta-analysis reported that the prevalence of PH is 5%–14% among patients with SSc (120). Identifying biomarkers that can predict the presence and progression of PH is crucial because it worsens the prognosis of patients with SSc (121). Izumiya et al. found that five let-7 family members (let-7a, -7d, -7e, -7f,and -7g) were downregulated in six PH skin tissues obtained from patients with SSc; let-7b and -7d expression levels were significantly and negatively correlated with the severity of PH in patients with SSc, indicating that skin miRNA levels could be a useful marker for predicting the presence and severity of PH in patients with SSc (117).

### lncRNA

Four lncRNAs associated with skin biology (122)—ANCR, TINCR, HOTTIP, and SPRY4-IT1 —have been identified as novel candidate biomarkers for SSc. Recent evidence suggests that TINCR, HOTTIP, and SPRY4-IT1 are upregulated, whereas ANCR is downregulated in plasma of patients with SSc compared with HCs. SPRY4-IT1 was significantly overexpressed in patients with dcSSC than in those with lcSSc, indicating its potential in the diagnosis of subtypes. SPRY-IT1, HOTTIP, and ANCR were also correlated with mRSS: ANCR expression was related to disease duration, the presence of PH, and altered lipid profile in patients with SSc, TINCR was positively correlated with ESR; and SPRY4-IT1 was associated with the presence of PH (118). Wang et al. found upregulated levels of lncTSIX in the serum of patients with SSc and fibroblast-conditioned medium, suggesting that dermal fibroblasts release and increase TSIX levels in patients with SSc. Elevated serum TSIX levels are a potential SSc biomarker as they are related to a significantly greater dcSSc:lcSSc ratio and higher mRSS (62).

## Conclusion

The high mortality and difficult diagnosis of SSc burden patients and society. Currently, next-generation sequencing technologies and other high-throughput methods can be used to readily detect the different expression levels of miRNAs and lncRNAs in healthy and disease conditions. Normally, the levels of miRNAs and lncRNAs are stable owing to the physiological balance between synthesis and degradation. However, dysregulated miRNA or lncRNA expression changes their functions and may promote the onset of SSc. In this review, we elaborated on the propathogenesis and antipathogenesis mechanisms of ncRNAs (miRNAs and lncRNAs) in the different stages of SSc, including microvascular changes, inflammation, and fibrosis. Several databases such as miRBase and starBase can be used to predict the functions of miRNAs and lncRNAs and their interactions with targeted proteins. Research on miRNAs and lncRNAs in SSc has focused on the transcriptional and post-transcriptional levels rather than post-translation level. Hence, further research on post-translational modifications is warranted to better understand the pathogenesis of autoimmune diseases. Recent studies have shown that some miRNAs and lncRNAs are highly expressed in the blood, indicating their potential value as noninvasive biomarkers for the diagnosis and prognosis of SSc. Compared with traditional proteins markers, ncRNAs appear more precise and sensitive, although their practicality is yet to be tested. Moreover, miRNAs may represent therapeutic targets to inhibit key pathogenic molecules and switch phenotypic changes involved in the development of SSc *via* siRNA, AS oligonucleotides, or CRISPR–Cas9-mediated genome editing. However, few applications of miRNAs for SSc-targeted therapy exist. Advances described in this review may stimulate additional research necessary to better understand the basic biology of miRNAs and lncRNAs and explore their potential as tools for clinical application in cases of SSc and other autoimmune diseases.

## Author Contributions

YZL and YML provided direction and guidance throughout the preparation of this manuscript. HZ, HL, XL, and YH collected and prepared the related literature. YML drafted the manuscript. YZL, LC, HZ, and HL reviewed and made significant revisions to the manuscript. All authors contributed to the article and approved the submitted version.

## Funding

This work was supported by the National Key Research and Development Program of China (2018YFE0207300), the National Natural Science Foundation of China (81871302), Beijing Municipal Science & Technology Commission (Z211100002521021).

## Conflict of Interest

The authors declare that the research was conducted in the absence of any commercial or financial relationships that could be construed as a potential conflict of interest.

## Publisher’s Note

All claims expressed in this article are solely those of the authors and do not necessarily represent those of their affiliated organizations, or those of the publisher, the editors and the reviewers. Any product that may be evaluated in this article, or claim that may be made by its manufacturer, is not guaranteed or endorsed by the publisher.

## Abbreviations

AGO, Argonaute; BMSC-EVs, bone marrow mesenchymal stem cell -derived extracellular vesicles; circRNAs, circular RNAs; CENPE, centromere protein F; Col1A2, collagen-typeIalpha 2; Col4A1, collagen-typeIValpha 1; CTGF, connective tissue growth factor; DDIT4L, DNA damage-inducible transcript 4-like protein; ECM, excessive extracellular matrix; EZH2, enhancer of zeste 2; FAP, fibroblast activation protein; HPASMCs, human pulmonary artery smooth muscle cells; IFN, interferon; IRF5, IFN regulatory factor 5; mRNA, message RNA; miRNAs, microRNAs; mRSS, modified Rodnan skin score; m7G, 7-methyl guanosine; lncRNAs, long non-coding RNAs; ncRNAs, non-coding RNAs; ncSSc, non-cutaneous SSc; NXF1, nucleus RNA export factor 1; PBMCs, peripheral blood mononuclear cells; pDCs, plasmacytoid dendritic cells; PDGF, platelet-derived growth factor; piRNAs, piwi-interacting RNAs; pri-miRNA, primary miRNA; RISC, RNA-induced silencing complex; STAT4, signal transducer and activator of transcription 4; SMILR, Smooth muscle enriched long non-coding RNA; SSc, systemic sclerosis; lcSSc, limited cutaneous SSc; dcSSc, diffuse cutaneous SSc; TGFBR2, TGF-β receptor types 2; TIMP-1, tissue inhibitor of metalloproteinases 1; tsRNAs, transfer RNA-derived small RNAs; TRBP, transactivation response element RNA-binding protein; TLR-9, toll-like receptor 9; UTR, untranslated region; uPA, urokinase-type plasminogen activator; VSMC, vascular smooth muscle cell.
